# Molecular subtyping and functional characterization of gastric cancer using arginine metabolism-related genes

**DOI:** 10.3389/fcell.2025.1732490

**Published:** 2026-01-07

**Authors:** Yongfu Shao, Xuan Yu, Jianing Yan, Haotian Dong

**Affiliations:** 1 Department of Gastroenterology, the First Affiliated Hospital of Ningbo University, Ningbo, China; 2 Health Science Center, Ningbo University, Ningbo, China

**Keywords:** arginine metabolism-related genes, gastric cancer, immune microenvironment, molecular subtype, prognosis

## Abstract

**Background:**

Gastric cancer (GC) remains one of the most lethal malignancies worldwide due to its substantial heterogeneity, necessitating improved therapeutic strategies and prognostic tools. Recent studies have implicated dysregulated arginine metabolism in GC pathogenesis; however, its metabolic characteristics and clinical prognostic value are not fully understood.

**Methods:**

GC samples were classified into three molecular subtypes based on the expression profiles of arginine metabolism-related genes (ArMGs). Prognostic ArMGs were identified within each subtype, and a cross-dataset prognostic model was developed to assess its predictive value. The associations of the model with anti-tumor immunotherapy, tumor immune microenvironment, signaling pathways, and gene expression patterns were further explored. Key candidate genes were validated using quantitative polymerase chain reaction (qPCR) in GC tissues and cell lines, and their biological functions were investigated through functional assays.

**Results:**

Consensus clustering of nine ArMGs stratified GC into three molecular subtypes: C1, C2, and C3. A prognostic prediction model for GC was constructed using differentially expressed genes among the subtypes and seven key prognostic genes: TMEM171, SLC5A1, DEGS2, MGP, C7, HMGCS2, and CREB3L3. The model demonstrated varying sensitivities to anti-tumor immunotherapy and showed strong correlations with immune-related tumor markers, the tumor immune microenvironment, and multiple signaling pathways. Among the ArMGs, ODC1 and ALDH18A1 were identified as critical contributors to GC. qPCR confirmed their elevated expression in GC tissues and cell lines. Silencing these genes significantly reduced GC cell proliferation, colony formation, and invasion.

**Conclusion:**

This study comprehensively characterized the molecular features of ArMGs in GC and developed a robust, validated prognostic prediction model. The findings offer new molecular insights for predicting patient outcomes and guiding personalized therapeutic strategies in GC.

## 1 Introduction

Gastric cancer (GC) is a highly complex malignant tumor of the digestive system characterized by diverse molecular phenotypes. It remains one of the leading causes of cancer-related deaths worldwide (Smyth et al., 2020). Epidemiologically, GC ranks fifth in incidence and fourth in mortality globally, posing a major threat to public health (Sung et al., 2021). The early symptoms of GC are often insidious and nonspecific, resulting in approximately 60%–70% of cases being diagnosed at intermediate or advanced stages. This delay significantly narrows the window for clinical intervention and contributes to poor prognosis (Wang and Wu, 2025; Mok et al., 2024). Despite advances in neoadjuvant chemotherapy and immunotherapy, the 5-year survival rate of patients with GC has remained largely stagnant over recent decades (Kim et al., 2025; Zhang X, et al., 2024). GC is characterized by substantial heterogeneity, with significant differences in biological behavior and clinical outcomes across pathological stages and molecular subtypes. Even among patients sharing the same TNM stage and histological features, treatment response and survival outcomes can vary significantly (Ho and Tan, 2019). The Glasgow Prognostic Score (GPS) has demonstrated utility in predicting outcomes for patients undergoing radical gastrectomy; however, its applicability remains limited in cases managed with conservative therapy, highlighting the limitations of conventional pathology-based prognostic tools (Majewska et al., 2025; Shimoda et al., 2022). Therefore, novel approaches are urgently needed to improve risk stratification and personalize GC treatment. Prognostic systems based on multi-omics data have emerged as a promising area of research. These models allow for dynamic monitoring of the tumor microenvironment (TME) and enable treatment sensitivity prediction. When integrated with machine learning, features such as gene expression in key signaling pathways, DNA methylation patterns, and immune infiltration scores can significantly improve survival prediction for patients receiving conservative treatment (Zeng et al., 2020; Chen et al., 2021; Tong et al., 2022). Such developments provide a theoretical foundation for GC-based precision medicine.

Tumorigenesis, proliferation, invasion, and metastasis are closely linked to alterations in the TME (De Visser and Joyce, 2023). Changes in cellular metabolism are a hallmark of cancer, as tumor-derived metabolites can impair immune cell activation and suppress anti-tumor responses, thereby promoting tumor progression. Metabolic reprogramming also enables immune evasion (Zhang S, et al., 2024; Yan et al., 2024). Metabolites from abnormal tumor pathways, such as the Warburg effect, influence immune cell recruitment and function within the TME, impairing CD8^+^ T cells and NK cell activity and fostering an immune-permissive environment for tumor growth (Brand et al., 2016; Yang et al., 2016). Notably, aberrant expression of metabolism-associated genes (MAGs) has been linked to oncogene dysregulation and demonstrates strong prognostic relevance in breast cancer and lymphoma (Tharp et al., 2024; He et al., 2022). With the increasing application of integrated multi-omics approaches, the relationship between metabolic pathways and prognosis is being more comprehensively explored. For example, Du et al. developed a metabolism-based multi-gene risk prediction model for thyroid cancer, capable of stratifying patients by prognosis and immune features, with potential relevance across various cancer types (Du et al., 2022). However, research on GC remains largely limited to studies of individual metabolites or single metabolic pathways (e.g., glycolysis or fatty acid oxidation), lacking comprehensive multi-omics network analyses and translatable prognostic models that capture the systemic interplay of arginine metabolism with the tumor immune microenvironment. This study addresses this critical gap by constructing an integrative model that links arginine metabolism gene signatures with immune profiles and clinical outcomes. In contrast to existing metabolism-focused models which often center on energy production or biomass synthesis, our model uniquely highlights arginine as a nexus of immunometabolic reprogramming, providing a novel framework for predicting immunotherapy response and patient stratification in GC.

As essential metabolic substrates, amino acids participate in protein synthesis and regulate cell fate through pathways involving nucleotide biosynthesis, redox balance, and epigenetic modifications (Neinast et al., 2019; Zhang et al., 2008; Ryan et al., 2021; Li and Zhang, 2024). They also serve as critical nutrients for immune and tumor cells (Sinclair et al., 2013; Li et al., 2020). Supplementation with free amino acids or modulation of transporter proteins has shown promise in inhibiting tumor growth and enhancing anti-tumor activity (Cao et al., 2016; Wu et al., 2021). Among amino acid metabolic pathways, arginine metabolism has attracted considerable attention for its immunomodulatory functions. For instance, arginase (ARG1) and nitric oxide synthase (NOS2) influence macrophage polarization: M1 macrophages metabolize arginine to nitric oxide (NO) via NOS2 to exert anti-tumor effects, whereas M2 macrophages convert arginine to urea, polyamines, and ornithine through ARG1 in response to tumor stimuli (Yerushalmi et al., 2006; Ou et al., 2023). Additionally, arginine contributes to nitrogen balance via the urea cycle, and its metabolite ornithine plays a central role in metastasis-associated biosynthetic pathways (Fung et al., 2025; Sun et al., 2024). Altered arginine metabolism has been specifically linked to GC progression. Downregulation of arginine succinate synthase 1 (ASS1) disrupts arginine homeostasis in the TME, leading to autophagy and growth suppression of GC cells (Tsai et al., 2018). Arginine deprivation therapy has shown promise in clinical trials targeting specific tumor types (Chu et al., 2023). However, comprehensive molecular models that integrate metabolic enzymes, metabolites, and immune context to characterize arginine metabolism in GC are still lacking.

This study aimed to systematically examine the expression and clinical relevance of ArMGs in GC. Initially, nine ArMGs associated with GC were selected using Gene Set Enrichment Analysis (GSEA) and data from The Cancer Genome Atlas (TCGA). Based on consensus clustering, GC samples were categorized into three molecular subtypes: C1, C2, and C3. Using differentially expressed genes among the subtypes, a prognostic risk model was constructed with Lasso-Cox regression and evaluated for predictive performance. Patients were stratified into high-risk and low-risk groups according to the model-derived risk score. The association of the model with anti-tumor immunotherapy response, immune-related tumor markers, immune microenvironment characteristics, and signaling pathways was further investigated. A nomogram was developed to enhance clinical applicability. Additionally, FeaturePlot was used to visualize gene expression in distinct cell populations, supporting model validation. Analysis of correlations among model genes, ArMGs, and immune-metabolic pathways led to the identification of ODC1 and ALDH18A1 as key candidate genes. These genes were validated via PCR in GC tissues and cell lines, and their functional roles were confirmed through *in vitro* assays. Overall, this study elucidated the molecular features of arginine-related prognostic genes in GC and established a validated prediction model, offering a theoretical basis for improved prognostic assessment and personalized therapy.

## 2 Materials and methods

### 2.1 Data preparation and processing

Gene expression data for stomach adenocarcinoma (STAD) were obtained from The Cancer Genome Atlas (TCGA, https://portal.gdc.cancer.gov/), comprising expression profiles from 448 patients. The GSE26901 Series Matrix File was downloaded from the National Center for Biotechnology Information (NCBI) Gene Expression Omnibus (GEO) database (https://www.ncbi.nlm.nih.gov/geo/info/datasets.html), with the annotation file being GPL6947, and included expression data from 109 patients. Similarly, the GSE62254 Series Matrix File was downloaded from GEO, annotated with GPL570, and contained expression data from 300 patients. In addition, single-cell RNA sequencing data from GSE183904 were retrieved from GEO, encompassing 36 samples with complete single-cell expression profiles, including 10 controls and 26 tumor samples.

### 2.2 Consistency clustering analysis

Gastric cancer subtypes were classified through consensus clustering based on the expression profiles of ArMGs. The clustering process was repeated 50 times, with 90% of the samples included in each iteration. The optimal number of clusters was determined by locating the inflection point in the cumulative distribution function curve of the consensus score.

### 2.3 Gene differential expression analysis

Differential expression analysis among the three sample groups was conducted using the R package “Limma”. Differentially expressed genes (DEGs) were identified with the thresholds of *P* < 0.05 and |log_2_FC| > 0.585. Volcano plots and heatmaps were generated to visualize the DEGs.

### 2.4 Model construction and prognosis

Intersecting genes were used to construct a prognostic model using the Lasso regression. A risk score was calculated for each patient by weighting the expression values of the selected genes with their corresponding Lasso regression coefficients. Patients were stratified into low-risk and high-risk groups based on the median risk score. Survival differences between the two groups were assessed using Kaplan-Meier analysis and compared using the log-rank test. The predictive value of the risk score was further evaluated using Lasso regression and stratified analyses. The accuracy of the model was examined using receiver operating characteristic (ROC) curve analysis.

### 2.5 Construction of the nomogram model

A points-based scoring system was developed using a multivariate regression model. Points were assigned to each level of the influencing factors according to the magnitude of their regression coefficients, reflecting their contribution to the outcome variable. The total score for each patient was obtained by summing these points, which allowed the prediction of the clinical outcome (Cheng et al., 2024).

### 2.6 Drug sensitivity analysis

The chemotherapeutic sensitivity of each tumor sample was predicted using the R package “oncoPredict”, with reference to the Genomics of Drug Sensitivity in Cancer (GDSC) database (https://www.cancerrxgene.org/). A regression approach was applied to estimate the IC50 values for each chemotherapy drug, and the GDSC training set was used for 10-fold cross-validation to assess regression and prediction accuracy. All analyses were conducted with default parameters, including the ComBat algorithm for batch effect correction and averaging of expression values for duplicate gene symbols.

### 2.7 Analysis of TMB, MSI, and NEO data

In this study, tumor mutational burden (TMB) was calculated as the mutation frequency, defined by the ratio of the number of variants to the exon length for each tumor sample. Microsatellite instability (MSI) scores for TCGA patients were obtained from a previously published study (Bonneville et al., 2017). Patient-specific neoantigens were predicted using NetMHCpan v3.0 (Huang and Fu, 2019).

### 2.8 Immune cell infiltration analysis

The CIBERSORT algorithm was used to estimate the relative proportions of 22 immune cell types within the tumor microenvironment based on patient gene expression data. Correlation analyses were then performed to evaluate associations between gene expression levels and immune cell abundancies.

### 2.9 GSVA

Gene sets were obtained from the Molecular Signatures Database (MSigDB, version 7.0). GSVA was performed to calculate enrichment scores for each gene set in individual samples, enabling the evaluation of potential biological functional differences across sample groups.

### 2.10 GSEA

Patients were stratified into high-risk and low-risk groups based on the prognostic model. GSEA was then conducted to identify signaling pathways that were differentially enriched between the two groups. Background gene sets for pathway annotation were sourced from the MSigDB database. Significantly enriched gene sets (adjusted *P* < 0.05) were ranked according to their enrichment scores. GSEA was employed to link tumor classification with underlying biological functions.

### 2.11 Single-cell analysis

The single-cell RNA sequencing data were first preprocessed and normalized using the Seurat package. The t-distributed stochastic neighbor embedding (t-SNE) algorithm was then applied to visualize the spatial distribution of distinct cell clusters. Cell types within the tissues were annotated based on known marker genes retrieved from the CellMarker database and supported by published literature.

### 2.12 Cell culture and transfection

Human GC cell lines (AGS and HGC-27) were obtained from the Cell Bank of the Chinese Academy of Sciences or the Shanghai Institute of Biochemistry and Cell Biology. Cells were cultured in RPMI-1640 medium (Invitrogen, Grand Island, USA) supplemented with 10% fetal bovine serum (FBS) and maintained at 37 °C in a humidified atmosphere with 5% CO_2_.

Actively growing cells were digested and seeded into 6-well plates. Once cells reached approximately 50%–60% confluence, transfection was performed according to the manufacturer’s instructions using Lipofectamine 2000 reagent (Invitrogen, CA, USA) and Opti-MEM I reduced-serum medium (Gibco, CA, USA). Cells were transfected with either siRNA (100 nM) or negative control siRNA (si-NC, 100 nM). After 6 h of transfection, the medium was replaced with RPMI-1640 supplemented with 10% FBS.

### 2.13 Clinical specimen collection

A total of 30 paired gastric cancer tissues and adjacent normal tissues were collected from surgical patients at the First Affiliated Hospital of Ningbo University from 2022 to 2024. None of the patients had undergone radiotherapy or chemotherapy prior to surgery, and all cancer tissues were confirmed by pathological examination. This study was performed in accordance with Chinese clinical guidelines and relevant regulations, and received approval from the ethics committee of the First Affiliated Hospital of Ningbo University (IRB No. KY20220101). Written informed consent was obtained from all patients.

### 2.14 RNA extraction, reverse transcription, and PCR detection

Total RNA was extracted using TRIzol Reagent (Thermo Fisher Scientific, USA). The extracted RNA was reverse transcribed into complementary DNA (cDNA) using a commercially available reverse transcription kit. Then, quantitative PCR was performed using the GoTaq qPCR master mix (Promega). Thermal cycling conditions included initial pre-denaturation at 95 °C for 5 min, followed by 40 cycles of denaturation at 94 °C for 15 s, annealing at 55 °C for 30 s, and extension at 72 °C for 30 s. Glyceraldehyde 3-phosphate dehydrogenase (GAPDH) was used as the internal control. Relative gene expression was quantified using the ΔCt method, where higher ΔCt values indicate lower expression levels. The primer sequences for the housekeeping gene GAPDH and the key genes ODC1 and ALDH18A1 are listed below: GAPDH(F): 5ʹ-ACCCACTCCTCCACCTTTGAC-3ʹ, (R): 5ʹ-TGTTGCTGTAGCCAAATTCGTT-3ʹ. ODC1(F): 5ʹ-TTTACTGCCAAGGACATTCTGG-3ʹ. (R): 5ʹ-GGAGAGCTTTTAACCACCTCAG-3ʹ. ALDH18A1(F): 5ʹ-GCCCTTCAACCAACATCTTCT-3ʹ. (R): 5ʹ-AGGGGTACAGTGATAAACGGG-3ʹ.

### 2.15 Cell proliferation assay

After 24 h of cell transfection, cells were seeded into 96-well plates at a density of 1 × 10^4^ cells per well. At 0, 24, 48, 72, and 96 h of incubation, 10 µL of CCK-8 reagent (Beyotime, Shanghai, China) was added to each well and incubated at 37 °C for 1 h. Optional density (OD) at 450 nm was measured using a multifunctional microplate reader (Thermo Fisher Scientific, MA, USA). Each sample was tested in six replicates.

### 2.16 Plate colony formation assay

After 24 h of cell transfection, cells were trypsinized into single-cell suspensions and seeded into 6-well plates at a density of 600 cells per well. After 14 days of incubation, each well was washed twice with phosphate-buffered saline (PBS), fixed with 4% paraformaldehyde (Beyotime, Jiangsu, China) for 20 min, and stained with 0.1% crystal violet solution (Beyotime, Jiangsu, China) for 20 min. The wells were then rinsed with double-distilled water until excess crystal violet was removed. Finally, the wells were photographed, and the number of stained colonies was counted. Each assay was performed in triplicate.

### 2.17 Transwell invasion assays

Transwell invasion assays were performed using transwell chambers (Corning, NY, USA) equipped with 8.0 μm-pore polycarbonate membranes coated with Matrigel (100 µg/mL; BD, NJ, USA). After 24 h of cell transfection, cells were resuspended in serum-free RPMI-1640 medium at a concentration of 1 × 10^5^ cells/mL. A total of 500 µL of RPMI-1640 medium containing 40% FBS was added to the lower chamber, while 200 µL of the cell suspension was added to the upper chamber. After incubation for 24–48 h at 37 °C, the membranes were washed with PBS, and the cells on the lower surface were fixed with paraformaldehyde for 20 min and stained with 0.1% crystal violet solution for 20 min. Each assay was performed in triplicate.

### 2.18 Statistical analysis

All statistical analyses were performed using R software (version 4.4.0) (https://www.r-project.org/) or Statistical Package for the Social Sciences (SPSS) 19.0. A *P-*value < 0.05 was considered statistically significant.

## 3 Results

### 3.1 Differential expression of ArMGs in GC tissues

**FIGURE 1:**
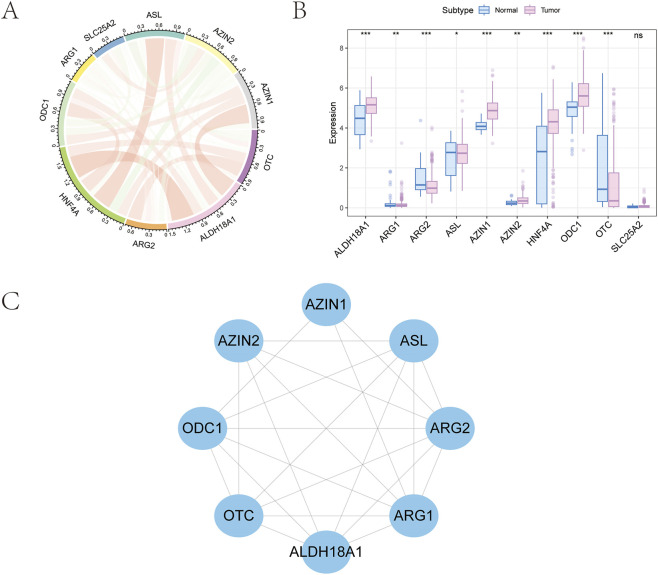
Selection of arginine metabolism-related genes in GC. **(A)** Ten prognosis-related genes were identified from the GSEA database. **(B)** Expression profile of nine genes showing significant differences between tumor and normal tissues. **(C)** Protein-protein interaction network of the selected genes.

Ten ArMGs were initially retrieved from the GSEA database (https://www.gsea-msigdb.org/gsea/msigdb). A co-expression network was then constructed using gene expression profiles from GC tissues and visualized as a correlation circle plot (Figure 1A). Differential expression analysis between normal and GC tissues revealed significant dysregulation in nine genes: AZIN2, HNF4A, ARG1, ARG2, ASL, ODC1, OTC, AZIN1, and ALDH18A1 (Figure 1B). Finally, a protein-protein interaction (PPI) network for these nine differentially expressed genes was generated using the STRING database (https://string-db.org) and visualized in Cytoscape (Figure 1C).

### 3.2 Identification of GC subtypes based on ArMGs

**FIGURE 2:**
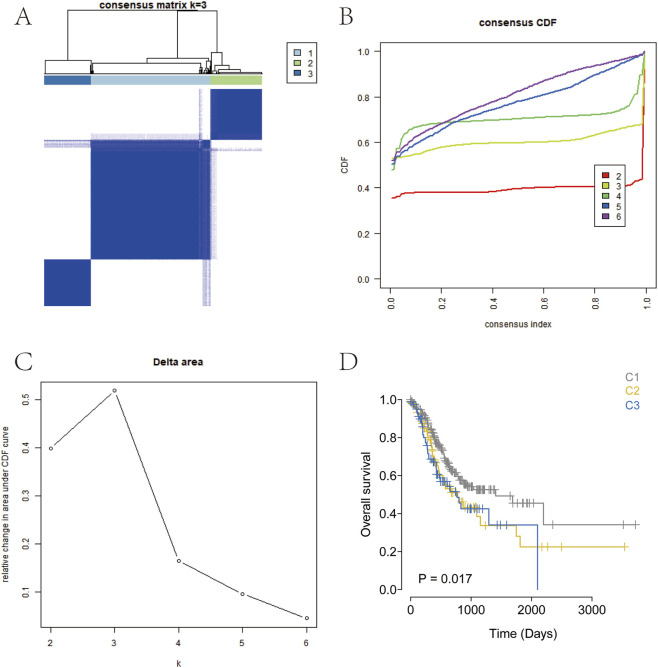
Consensus clustering for GC subtyping. **(A)** Consensus heatmap for k = 3. **(B)** Cumulative distribution function (CDF) curves from k = 2–7. **(C)** CDF delta area curves for k = 2–7. **(D)** Kaplan-Meier survival curve of the three GC subtypes.

Consensus clustering based on the expression levels of differentially expressed ArMGs was performed for the molecular classification of GC samples (Figures 2A–C). The results showed that when K = 3, the boundaries among the three subtypes were relatively distinct. Accordingly, the GC dataset was divided into three clusters: Cluster 1 (C1), Cluster 2 (C2), and Cluster 3 (C3). Subsequent survival analysis revealed statistically significant differences in overall survival among the subtypes (*P* < 0.05; Figure 2D). Notably, the C1 subtype exhibited a more favorable survival probability than the other two subtypes. The distinct arginine metabolism patterns observed among the C1, C2, and C3 subtypes likely arise from the convergence of upstream oncogenic signaling pathways and tumor microenvironmental pressures.

### 3.3 Identification of DEGs among GC subtypes

**FIGURE 3:**
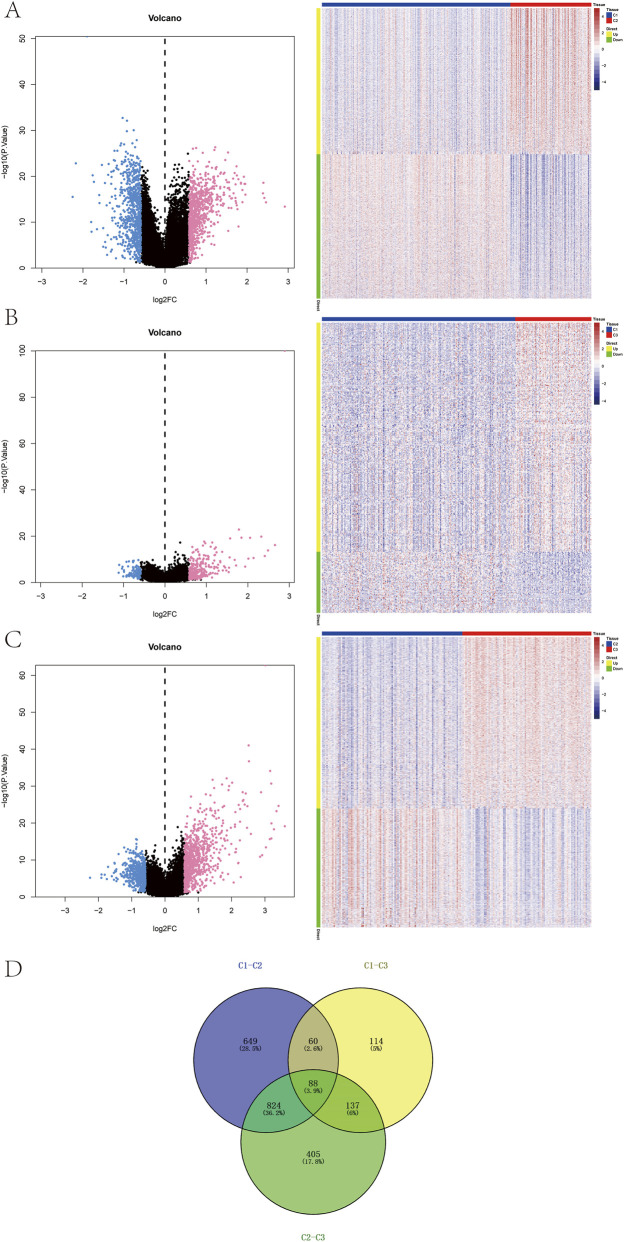
Identification of subtype-specific genes. **(A–C)** Volcano plots and heatmaps showing differentially expressed genes (DEGs) among the three subtypes. **(D)** Venn diagram of DEGs across subtypes.

Differential expression analysis was performed based on the subtype groupings using the limma package to identify DEGs between the subtypes. DEGs were selected according to the criteria of *P*-value < 0.05 and |logFC| > 0.585. Between the C1 and C2 groups, a total of 1,621 DEGs were identified, including 816 upregulated and 805 downregulated genes (Figure 3A). Between the C1 and C3 groups, 399 DEGs were detected, with 315 upregulated and 84 downregulated genes (Figure 3B). Between the C2 and C3 groups, 1,454 DEGs were identified, comprising 859 upregulated and 595 downregulated genes (Figure 3C). The intersection of these three DEG sets yielded 88 overlapping genes (Figure 3D).

### 3.4 Identification of prognostic genes and construction of the prognostic risk model

**FIGURE 4:**
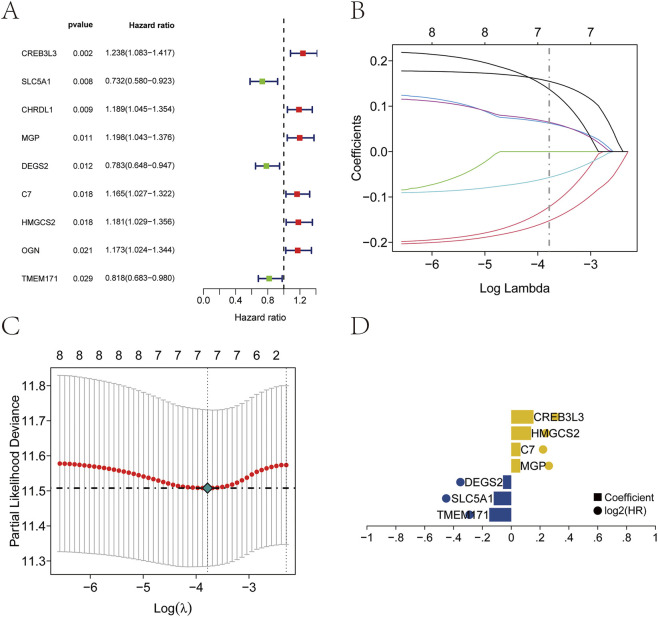
Establishment of the prognostic model. **(A)** Nine prognostic genes identified by univariate Cox regression. **(B–D)** Construction of the prognostic model using Lasso regression.

Clinical and survival data for patients with GC were obtained from the TCGA database. Univariate Cox regression analysis identified nine genes that were significantly associated with prognosis (*P* < 0.05; Figure 4A). Seven key prognostic genes were selected using the Lasso regression feature selection algorithm. The TCGA dataset was randomly divided into training and test sets at a ratio of 4:1. Lasso regression analysis was then applied to derive the model coefficients for each sample (Figures 4B–D). The optimal riskscore value was used for subsequent analysis. The risk score formula was defined as: RiskScore = TMEM171× (−0.152323115295076) + SLC5A1 × (−0.121599685780654) + DEGS2 × (−0.0569621957618535) + MGP × 0.0623411051770642 + C7 × 0.064629072356396 + HMGCS2 × 0.137353168245766 + CREB3L3 × 0.154874633934315.

### 3.5 Construction of the nomogram prediction model

**FIGURE 5:**
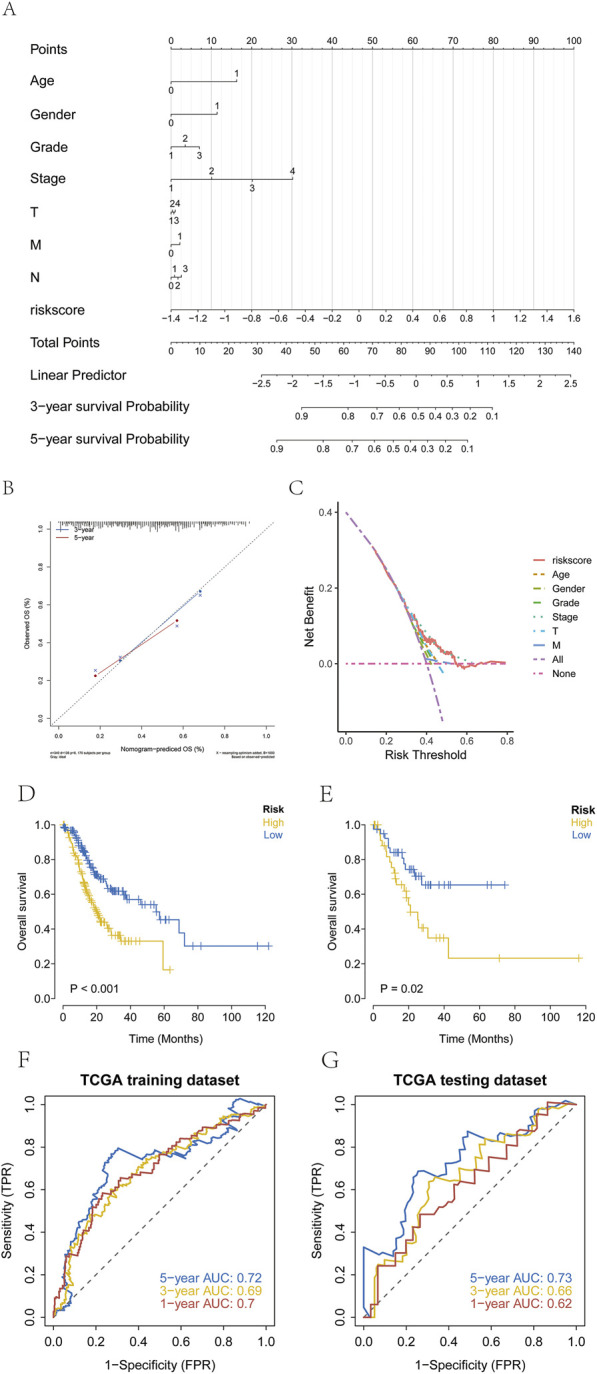
Establishment of risk groups. **(A–C)** Stratification of patients with GC into high-risk and low-risk groups using regression analysis, with prediction of three and 5-year survival and evaluation by decision curve analysis (DCA). (Age: 0=<60, 1 = ≥60; Gender: 0 = female, 1 = male; Grade: 1 = G1, 2 = G2, 3 = G3; Stage: 1 = Stage I, 2 = Stage II, 3 = Stage III, 4 = Stage IV; T: 1 = T1, 2 = T2, 3 = T3,4 = T4; N: 1 = N0; 2 = N1; 3 = N2, 4 = N3; M: 0 = M0, 1 = M1). **(D,E)** Kaplan-Meier survival curves showing significantly poorer OS in the high-risk group than in the low-risk group. **(F,G)** ROC curves validating model performance in the TCGA cohort.

Samples were stratified into high-risk and low-risk groups based on the median risk score. A nomogram was constructed to visually present the results of regression analysis. Logistic regression analysis revealed that the risk score significantly contributed to the predictive scoring process of the nomogram (Figure 5A). In the nomogram, the points scale assigns a score for each variable. For continuous variables (Age, Risk Score), higher original values correspond to higher points. The cut-off for Age (60 years) was determined based on the median age in our cohort and is commonly used in clinical stratification. Categorical clinical variables (Gender, T, N, M stage, Grade, Stage) are displayed with their actual clinical categories on the axis for clarity. Predictive analysis was performed for 3-year and 5-year overall survival (OS) in patients with GC (Figure 5B), and a decision curve analysis (DCA) was generated to evaluate clinical benefit (Figure 5C). Kaplan-Meier survival analysis demonstrated significantly shorter OS in the high-risk groups across both the training and test cohorts (Figures 5D,E). Receiver operating characteristic (ROC) curves further confirmed the predictive performance of the model (Figures 5F,G).

### 3.6 Validation of the prognostic risk model

**FIGURE 6:**
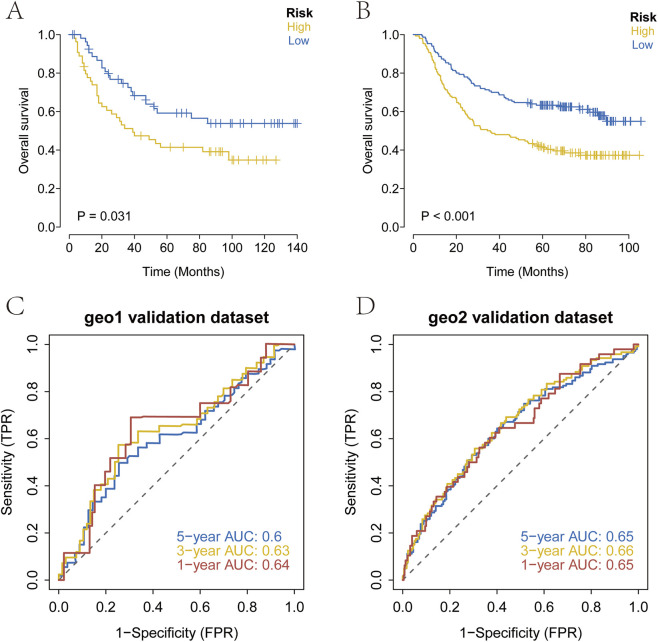
External validation of the prognostic model. **(A–D)** Kaplan-Meier survival curves and ROC curves in the GEO cohort. High-risk patients showed significantly poorer OS, and the ROC analysis confirmed strong predictive performance.

Processed transcriptomic datasets with complete survival information (GSE26901, GSE62254) were obtained from the GEO database for external validation. The established prognostic model was applied to stratify patients with GC into high-risk and low-risk groups. Kaplan-Meier survival analysis was used to evaluate survival differences between the two groups and assess the stability of the prediction model. The results indicated that OS in the high-risk group was significantly lower than that in the low-risk group within the GEO validation datasets (Figures 6A,B). To further verify the predictive accuracy of the model, ROC curve analysis was conducted using the external datasets. The results demonstrated that the model exhibited strong prognostic performance (Figures 6C,D).

### 3.7 Analysis of sensitivity to chemotherapy and immunotherapy

**FIGURE 7:**
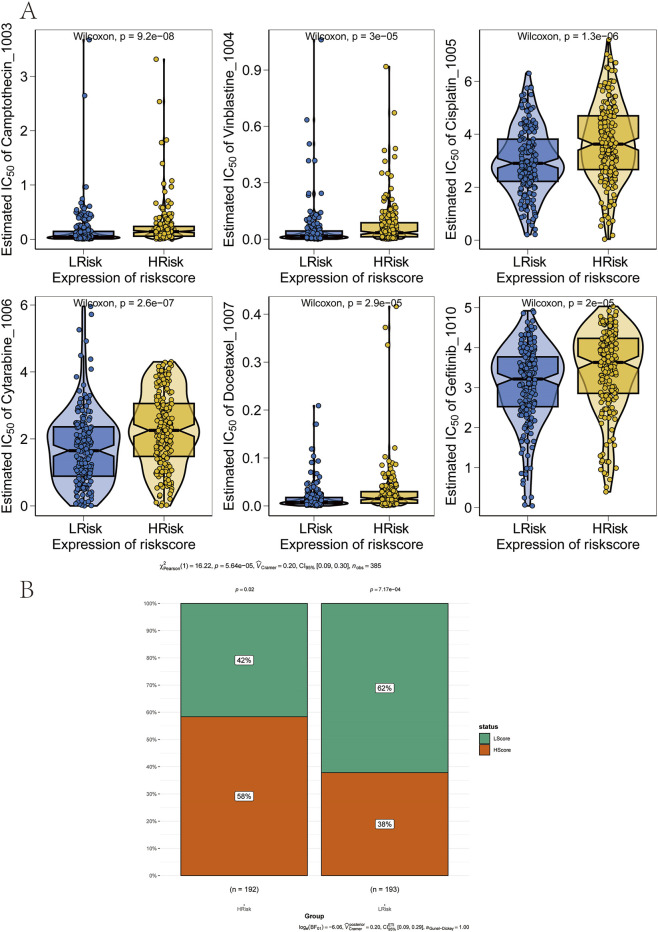
Association between the prognostic model and therapy response. **(A)** Correlation between risk groups and sensitivity to chemotherapy drugs. **(B)** Reduced sensitivity to immunotherapy in the high-risk group.

The therapeutic efficacy of surgery combined with chemotherapy for early-stage GC is well established. In this study, drug sensitivity data from the GDSC database were combined with the R package “oncoPredict” to estimate the chemotherapy sensitivity of each tumor sample. The relationship between risk score and sensitivity to common chemotherapeutic agents was further examined. The results indicated that the risk score was significantly associated with sensitivity to several drugs, including Camptothecin_1003, Vinblastine_1004, Cisplatin_1005, Cytarabine_1006, Docetaxel_1007, and Gefitinib_1010 (Figure 7A). In addition, immunotherapy response prediction based on the risk score was performed using the Tumor Immune Dysfunction and Exclusion (TIDE) algorithm. The TIDE score integrates expression signatures of T-cell dysfunction and exclusion to predict clinical response to immune checkpoint blockade therapies, primarily targeting the PD-1/PD-L1 and CTLA-4 axes. The results revealed that patients in the high-risk group exhibited higher TIDE scores, indicating a poorer predicted response to anti-PD-1/CTLA-4 immunotherapy (Figure 7B).

### 3.8 Correlation analysis of risk scores with genomic and immunological features of GC

**FIGURE 8:**
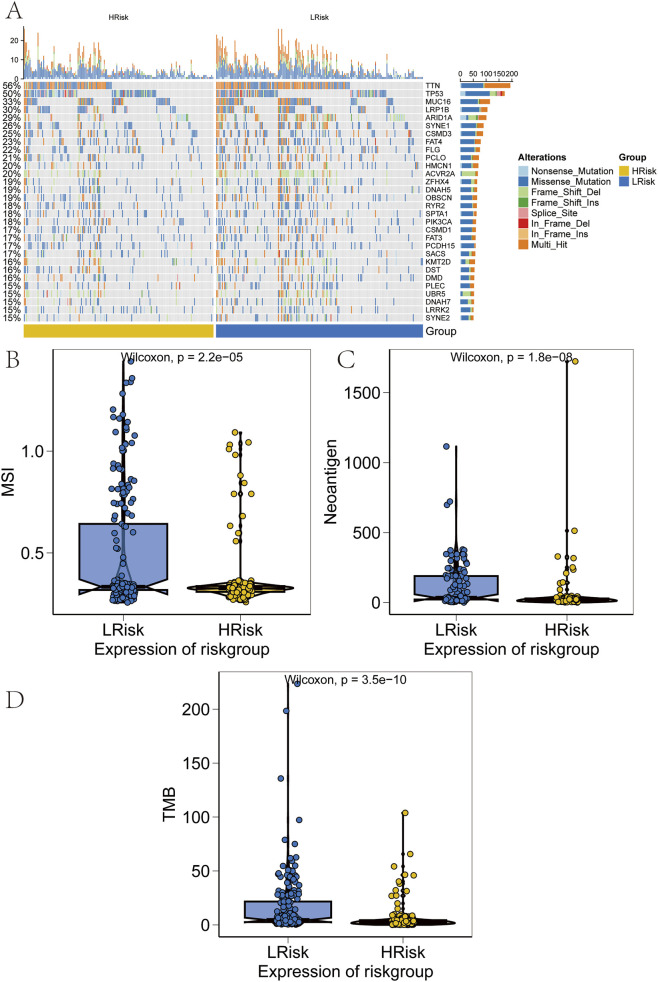
Validation of performance across risk groups. **(A)** Heatmap of somatic mutation spectra in high and low-risk groups, with percentage indicating mutation frequency. **(B–D)** Comparison of microsatellite instability (MSI), neoantigen (NEO) load, and tumor mutational burden (TMB) between risk groups.

SNP-related data for processed GC samples were downloaded and analyzed. The 30 most frequently mutated genes were selected to compare mutation patterns between high-risk and low-risk patient subgroups. Mutation frequency differences were visualized using the R package “ComplexHeatmap” (Figure 8A). Notably, the mutation frequencies of TTN and several other genes were significantly lower in the high-risk group than in the low-risk group. Next, associations between the prognostic risk score and established immunotherapy biomarkers were assessed. Significant differences were observed between high-risk and low-risk groups in epithelial–mesenchymal transition (EMT) scores, microsatellite instability (MSI), tumor mutation burden (TMB), and tumor neoantigen (Figures 8B–D).

### 3.9 Correlation analysis of risk scores with immune cell infiltration

**FIGURE 9:**
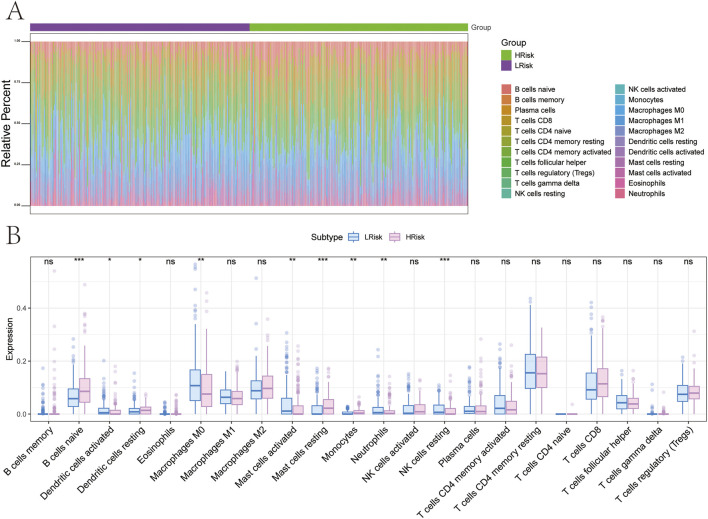
Relationship between different risk groups and immune cell infiltration. **(A,B)** Differences in immune cell composition between high and low-risk groups.

The tumor microenvironment, consisting of cancer-associated fibroblasts, immune cells, extracellular matrix components, signaling molecules, and malignant cells, plays a crucial role in tumor diagnosis, prognosis, and therapeutic response. To investigate the molecular mechanisms linking risk scores to GC progression, correlations between risk scores and immune infiltration profiles were assessed. Comparative analysis between the high-risk and low-risk subgroups revealed significant differences in immune cell composition (Figure 9A). In the high-risk group, decreased infiltration of dendritic cells activated, macrophages M0, mast cells activated, neutrophils, and natural killer (NK) cells resting was observed. Conversely, higher levels of B cells naive, dendritic cells resting, mast cells resting, and monocytes were detected (Figure 9B).

### 3.10 Analysis of the differences in signaling pathways between the risk groups

**FIGURE 10:**
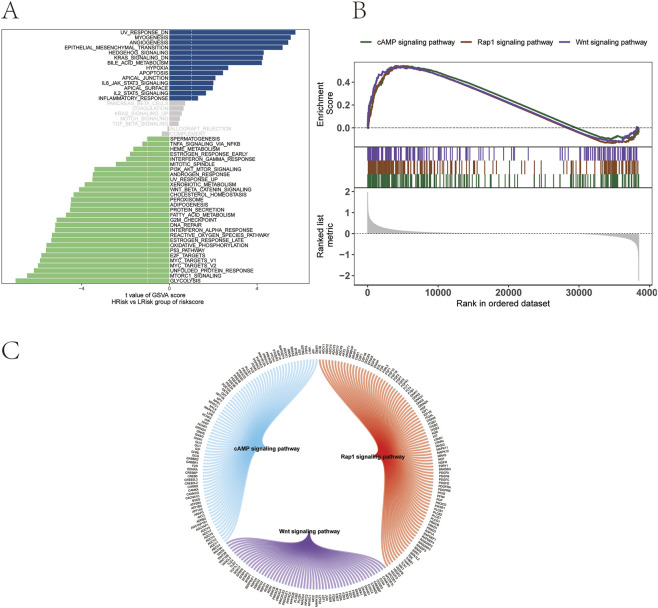
Functional pathway analysis of risk groups. **(A)** GSVA. **(B)** GSEA. **(C)** Molecular interaction network of enriched signaling pathways.

To further explore the signaling pathways associated with high and low-risk groups, the molecular mechanisms by which risk scores may influence tumor progression were investigated. GSVA revealed that differential pathways between the two groups were primarily enriched in HEDGEHOG_SIGNALING, IL6_JAK_STAT3_SIGNALING, and IL2_STAT5_SIGNALING pathways (Figure 10A). GSEA identified additional pathways, including the cAMP signaling pathway, Rap1 signaling pathway, and Wnt signaling pathway (Figure 10B). The molecular interaction network among these pathways is shown in Figure 10C.

### 3.11 Single-cell analysis of the model genes

**FIGURE 11:**
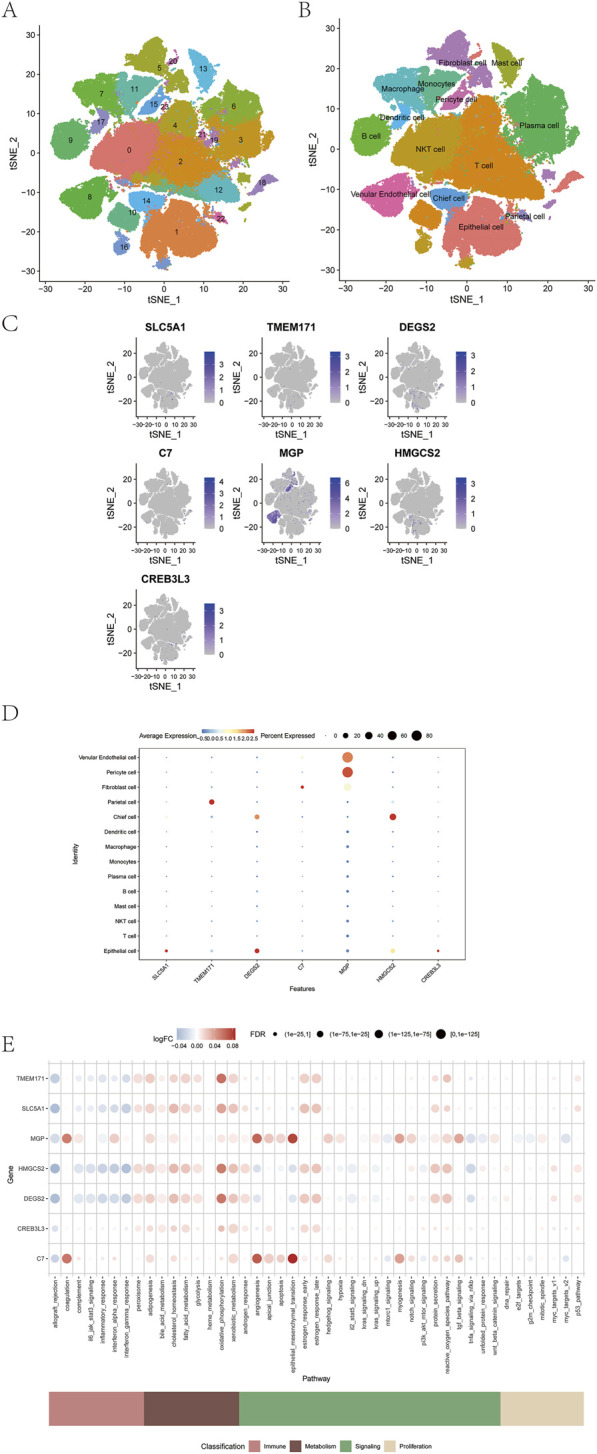
Correlation between prognostic model genes and tumor immunity. **(A–D)** Expression of seven prognostic model genes across immune cell types. **(E)** Correlation between model genes and immune metabolic pathways using AUCell analysis.

Single-cell transcriptomic analysis was performed using the t-SNE algorithm, resulting in the identification of 24 distinct cellular subtypes (Figure 11A). These subtypes were annotated into 14 major cell lineages: venular endothelial cells, pericytes, fibroblasts, parietal cells, chief cells, dendritic cells, macrophages, monocytes, plasma cells, B cells, mast cells, NKT cells, T cells, and epithelial cells (Figure 11B). The expression patterns of the seven prognostic model genes were visualized across all annotated lineages (Figures 11C,D). In addition, the AUCell function was used to perform quantitative analysis of immune-related and metabolic pathways at the single-cell level, demonstrating correlations between model gene expression and specific immune and metabolic signaling pathways (Figure 11E).

### 3.12 Verification of differential expression and biological function of key genes in GC

A comprehensive review of the literature on the nine ArMGs was conducted in the context of cancer research. Based on the co-expression network analysis between the model genes and ArMGs, ODC1 and ALDH18A1 were selected as target genes for further investigation.

**FIGURE 12:**
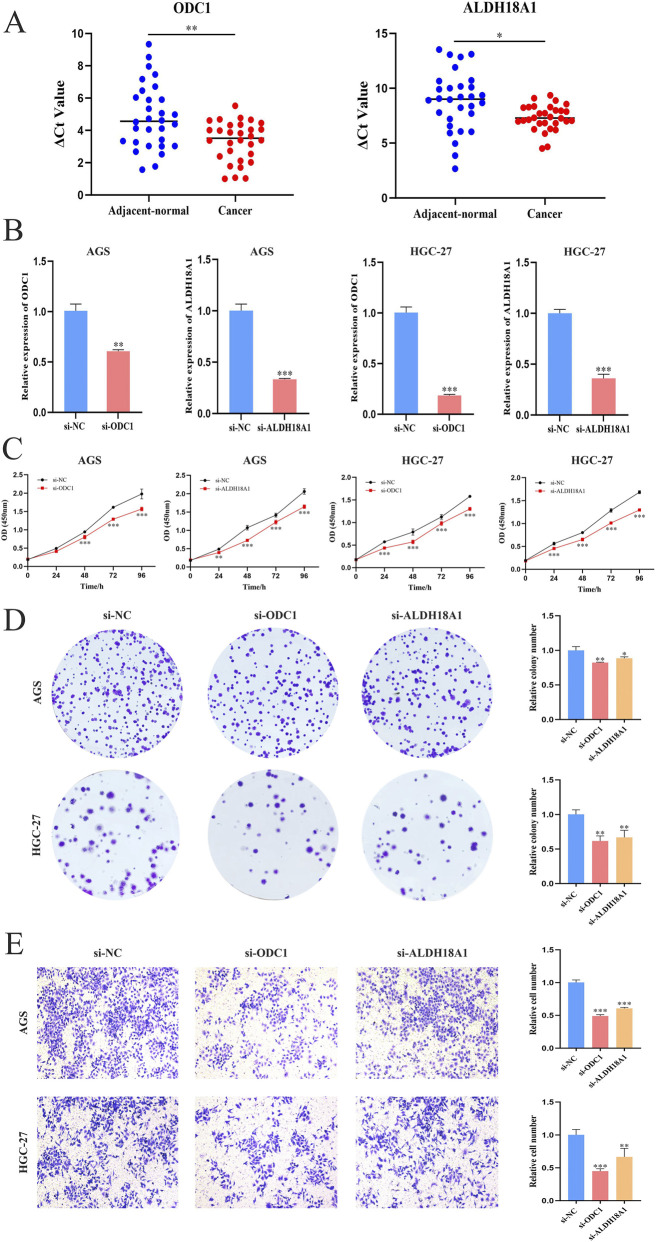
Validation of differential expression and biological functions of key genes in GC. **(A)** Expression levels of ODC1 and ALDH18A1 in GC and adjacent normal tissues (*n* = 30); lower ΔCt values indicate higher expression. **(B)** Verification of ODC1 and ALDH18A1 knockdown efficiency in AGS and HGC-27 cells by PCR. **(C)** CCK-8 assay showing reduced proliferation after knockdown (six replicates per sample). **(D)** Colony formation assay. **(E)** Transwell invasion assay. Functional assays consistently showed that silencing ODC1 or ALDH18A1 significantly inhibited GC cell proliferation, colony formation, and invasion (Student’s t-test, *P < 0.05, **P < 0.01, and ***P < 0.001).

Validation of ODC1 and ALDH18A1 expression and clinical relevance in GC was performed using cell lines and clinical tissue specimens. qPCR analysis revealed that both genes were significantly upregulated in GC tissues compared with adjacent normal tissues (Figure 12A). Two GC cell lines (AGS and HGC-27), which showed significant differential expression of the target genes relative to the normal gastric epithelial cell line GES-1, were selected for subsequent functional assays. To explore the biological roles of ODC1 and ALDH18A1 in GC progression, siRNAs targeting each gene were transfected into AGS and HGC-27 cells. The knockdown efficiency was confirmed by PCR (Figure 12B). The functional consequences of gene silencing were evaluated using cell proliferation, colony formation, and transwell invasion assays. Experimental results demonstrated that knockdown of ODC1 and ALDH18A1 significantly inhibited GC cell proliferation (Figure 12C), colony formation (Figure 12D), and invasive capacity (Figure 12E).

## 4 Discussion

GC is a common and aggressive malignancy of the digestive system that poses a serious threat to human health (Sung et al., 2021). Its development is a complex, multi-step process influenced by various factors, including *Helicobacter pylori* infection, genetic predisposition, high-nitrate diets, and environmental pollution (Smyth et al., 2020). Despite recent advances in endoscopic screening techniques, the early diagnosis rate of GC remains below 20%, and the 5-year survival rate for advanced-stage patients is less than 30% (Jeddi et al., 2018), underscoring the persistent challenges in clinical management. Traditional histopathological classification fails to adequately capture tumor heterogeneity, thereby limiting its utility in guiding personalized treatment and prognosis prediction. Moreover, current diagnostic methods lack reliability in predicting therapeutic response to emerging modalities such as immune checkpoint inhibitors and targeted therapies (Kang et al., 2017). In this context, metabolic reprogramming has emerged as a hallmark of cancer, with dynamic changes in metabolic gene expression closely associated with treatment responsiveness. For example, arginine-degrading enzymes have demonstrated synergistic anti-tumor effects when combined with oxaliplatin in colorectal cancer cells (Alexandrou et al., 2018). Therefore, metabolic profile-based prognostic models offer a promising alternative to conventional TNM staging for GC. Such models can monitor diverse metabolic states, enabling improved prognostic prediction and potentially enhancing patient survival outcomes.

Metabolic communication underpins essential physiological processes and significantly influences tumor proliferation, invasion, and cellular function (Su et al., 2024). Nearly all tumors exhibit aberrant metabolic pathways that contribute to malignant phenotypes (Li and Simon, 2020). Cancer cells can reprogram metabolic networks to satisfy increased energy, biosynthetic materials, and redox balance demands, thereby supporting tumor growth and survival (Martinez-Reyes and Chandel, 2021). Emerging evidence suggests that altered tumor metabolism also impairs immune cell function and reduces the efficacy of immunotherapy by reshaping the tumor microenvironment (Nenkov et al., 2021). Tumor cells deplete glucose via aerobic glycolysis and consume key amino acids (e.g., glutamine, serine, glycine, or branched-chain amino acids), thereby suppressing the function of T cells and NK cells and promoting immunosuppression (Kedia-Mehta and Finlay, 2019). Competition for nutrients and accumulation of inhibitory metabolites fundamentally alter the immune landscape of the TME (Tan et al., 2020). Given these observations, it is crucial to identify differentially expressed metabolic genes in GC tissues relative to normal epithelium. These genes may serve as molecular targets for precision therapy. As research into oncogene function and tumor pathogenesis advances, gene-based targeted therapy has become a cornerstone of treatment for advanced and recurrent GC. Therefore, identifying robust biomarkers and developing accurate molecular subtype classification systems are essential for improving prognostic assessment and guiding more personalized treatment approaches.

In this study, we performed consensus clustering based on nine ArMGs and identified three distinct molecular subtypes of GC. These subtypes exhibited significant differences in survival outcomes, with Cluster 1 showing the best prognosis and Cluster 3 showing the poorest (Figure 2D). Subsequent differential expression analysis across subtypes identified seven prognostic genes: TMEM171, SLC5A1, DEGS2, MGP, C7, HMGCS2, and CREB3L3. A prognostic risk model incorporating these seven genes was constructed using Lasso regression in the TCGA training cohort. Patients were stratified into high-risk and low-risk groups based on the median risk score. A nomogram integrating the risk score with relevant clinical features was developed to predict three and 5-year survival (Figures 5A–C). The risk score served as an independent prognostic factor and demonstrated high predictive accuracy in both the TCGA test cohort (Figures 5D–G) and an external GEO validation cohort (Figure 6). ROC analysis confirmed the strong predictive power of the model. Multiple validation strategies confirmed the robustness and accuracy of this prognostic model, which outperformed individual clinical variables in predicting GC patient outcomes. These results underscore the potential utility of the model for personalized risk stratification, clinical decision-making, and precision therapy in GC.

Chemoresistance remains a major barrier to successful treatment and is a leading cause of therapeutic failure in GC (Che et al., 2024). To address this, the association between risk scores and sensitivity to commonly used chemotherapeutic agents was investigated. Notably, patients in the high-risk group demonstrated significantly reduced sensitivity to drugs such as Cisplatin, Cytarabine and Gefitinib compared to those in the low-risk group, suggesting a higher likelihood of chemoresistance in the high-risk population (Figure 7A). Furthermore, elevated TIDE scores in the high-risk group indicated a higher probability of immune escape and immune dysfunction, which mechanistically explains a poorer response to immunotherapy (Figure 7B). These findings are consistent with recent literature, further validating the prognostic relevance of the risk score model. Given the established link between metabolic reprogramming and the tumor immune microenvironment, additional immune analyses were conducted to delineate the immune landscape across risk groups. The high-risk group exhibited lower immune scores and reduced immune activity, which were correlated with poorer survival outcomes, while the low-risk group showed a more favorable immune profile. Additionally, TMB, MSI, and NEO were examined, all of which showed negative associations with the risk score (Figure 8). These results further highlight the close relationship between the risk score, immune evasion, and sensitivity to immune checkpoint inhibitor (ICI) therapies, reinforcing its predictive strength. Collectively, the risk score demonstrated significant associations not only with immune cell infiltration and chemotherapeutic sensitivity but also with immunogenic biomarkers, including MSI, TMB, and NEO.

To further elucidate the molecular mechanisms underlying tumor progression in high-risk and low-risk patients, GSVA and GSEA analyses were conducted. These findings revealed the enrichment of multiple oncogenic signaling pathways, including those related to cell cycle regulation and apoptosis (Figures 10A,B). The results demonstrated that the model genes interact with cell cycle and cell death pathways through multiple signaling mechanisms, thereby contributing to GC initiation and progression. These findings provide a valuable direction for further molecular research. Furthermore, two key genes from the arginine metabolism gene set, ODC1 and ALDH18A1, were identified for in-depth investigation to clarify their roles in the GC prognostic model and the associated molecular network mechanisms. ODC1, a rate-limiting enzyme in the polyamine metabolic pathway (Casero et al., 2018), has been widely reported to be closely associated with tumor progression and poor prognosis across multiple malignancies. For example, in liver and prostate cancers, elevated ODC1 expression promotes polyamine accumulation, thereby significantly enhancing cell proliferation and invasion (Zhang H, et al., 2024; Kaminski et al., 2019). ODC1 is often regarded as an invasive phenotype biomarker. Similarly, ALDH18A1, a key enzyme in the *de novo* synthesis of proline and arginine, plays a crucial role in tumor metabolic reprogramming (Hu et al., 2008; Liu et al., 2015), Previous studies have shown that ALDH18A1 promotes the proliferation of hepatocellular carcinoma by influencing glycolysis and the pentose phosphate pathway (Ding et al., 2020). Moreover, silencing ALDH18A1 under hypoxic conditions prolongs the overall survival of patients with glioblastoma (Fang et al., 2019). In the context of GC, both ODC1 and ALDH18A1 were abnormally expressed in tumor cell lines, and their expression was associated with multiple signaling pathways linked to disease progression. Functional assays confirmed the oncogenic effects of these genes: targeted knockdown of ODC1 or ALDH18A1 expression significantly inhibited GC cell proliferation, colony formation, and invasion. These findings strongly support the roles of ODC1 and ALDH18A1 as driver genes in GC, providing a theoretical basis and new directions for the development of stratified diagnostic approaches and therapeutic strategies based on arginine metabolism and precision medicine.

This study established a prognostic model for GC based on arginine metabolism genes by integrating multi-omics data and highlighted the pivotal roles of ODC1 and ALDH18A1 in GC progression. Collectively, the prognostic model demonstrated significant associations not only with immune cell infiltration and chemotherapeutic sensitivity but also with immunogenic biomarkers. Importantly, as shown in our comparative analysis, this model provided superior prognostic discrimination and greater potential clinical net benefit compared to the widely used AJCC staging and Lauren classification, highlighting its promise as a complementary tool for refining risk stratification in GC. While this study establishes a promising prognostic model, several limitations should be acknowledged. First, the expression and biological functions of ODC1 and ALDH18A1 in tumor cells were confirmed through *in vitro* experiments, validation in several independent clinical samples has not yet been performed. Verification in diverse and large-scale clinical cohorts is essential to verify the robustness and generalizability of the model across different patient populations. Second, the prognostic model was developed using retrospective data from the TCGA database. While this dataset provides extensive molecular and clinical information, it may not fully capture the dynamic progression of GC or the heterogeneity of clinical manifestations. Therefore, prospective studies are required to further assess the clinical applicability of the model. Such studies can provide real-time data and enable the evaluation of predictive accuracy in clinical practice, thereby enhancing the translational potential of the model. Third, the model’s predictive power in early GC appears attenuated, likely due to the limited event numbers in this subgroup. Enrolling larger early-stage cohorts is needed to refine the model for early detection applications. Fourth, the generalizability across diverse ethnic populations requires verification. Addressing these limitations is critical for advancing the clinical utility of the GC prognostic model and ensuring its effectiveness in guiding individualized treatment strategies.

This study systematically characterized the molecular features of ArMGs in GC and developed a validated prognostic prediction model. The model not only accurately assessed survival risk in patients with GC but also demonstrated significant associations with immunotherapy sensitivity, immune-related tumor markers, the tumor immune microenvironment, and activation of signaling pathways. Among the identified genes, ODC1 and ALDH18A1 were identified as key regulators of GC progression. These findings provide novel molecular evidence for prognostic evaluation and offer valuable guidance for the development of individualized GC treatment strategies.

## Data Availability

The datasets presented in this study can be found in online repositories. The names of the repository/repositories and accession number(s) can be found below: https://www.jianguoyun.com/p/Db3zrc4Q6a7kDRia2pYGIAA, Jianguo Yun.
